# Inhibition of Angiogenesis and Extracellular Matrix Remodeling: Synergistic Effect of Renin-Angiotensin System Inhibitors and Bevacizumab

**DOI:** 10.3389/fonc.2022.829059

**Published:** 2022-07-01

**Authors:** Tianshu Ren, Hui Jia, Qiong Wu, Yan Zhang, Qun Ma, Dong Yao, Xudong Gao, Danni Xie, Zihua Xu, Qingchun Zhao, Yingshi Zhang

**Affiliations:** ^1^Department of Life Science and Biochemistry, Shenyang Pharmaceutical University, Shenyang, China; ^2^Department of Pharmacy, General Hospital of Northern Theater Command, Shenyang, China

**Keywords:** bevacizumab, renin-angiotensin system inhibitors, hypertension, angiogenesis, extracellular matrix components

## Abstract

Bevacizumab (Bev) is a humanized vascular endothelial growth factor monoclonal antibody that is used with chemotherapeutic drugs for the treatment of metastatic colorectal cancer (mCRC). Bev-induced hypertension (HT) is the most common adverse reaction during clinical practice. However, at present, appropriate antihypertensive agents for Bev-induced HT are unavailable. In this study, retrospective analysis of clinical data from mCRC patients who received renin-angiotensin system inhibitors (RASIs) showed significant survival benefits of overall survival (OS) and progression-free survival (PFS) over patients who received calcium channel blockers (CCBs) and patients who received no antihypertensive drug (NO: Y2020046 retrospectively registered). An experiment of HCT116 colon cancer cell xenografts in mice confirmed that combined treatment of Bev and lisinopril (Lis), a RASI, synergistically inhibited subcutaneous tumor growth and enhanced the concentration of 5-fluorouracil (5-Fu) in tumor tissues. Our results showed that the addition of Lis did not interfere with the vascular normalization effect promoted by Bev, but also inhibited collagen and hyaluronic acid (HA) deposition and significantly downregulated the expression of TGF-β1 and downstream SMAD signaling components which were enhanced by Bev, ultimately remodeling primary extracellular matrix components. In conclusion, RASIs and Bev have synergistic effect in the treatment of colorectal cancer and RASIs might be an optimal choice for the treatment of Bev-induced HT.

## Introduction

Bevacizumab (Bev) is a humanized vascular endothelial growth factor monoclonal antibody that is used with chemotherapeutic drugs, including 5-fluorouracil (5-Fu) or capecitabine, oxaliplatin or/and irinotecan-based chemotherapy, for the treatment of metastatic colorectal cancer (mCRC) (1–3). However, Bev-induced hypertension (HT), the most common adverse reaction, has an incidence rate exceeding 30%, which influences the process of anti-tumor treatment and causes cardiovascular events (4–7).

Guideline recommend when the systolic blood pressure ≥165 mm Hg or diastolic blood pressure ≥100 mm Hg, anti-angiogenesis therapy should be combined with anti-hypertensive agents and maintained until the blood pressure reaches to 140/90 mmHg (8). For the treatment of essential HT, thiazide diuretics, beta-adrenoceptor antagonists, calcium channel blockers (CCBs), angiotensin converting enzyme inhibitors (ACEIs) and angiotensin receptor blockers (ARBs) are widely used (9, 10). However, at present, appropriate antihypertensive agents for Bev-induced HT are unavailable, because patients undergoing chemotherapy and targeted therapy are excluded from clinical trials (11). Additionally, given the specific condition of patients with malignant tumors, the choice of antihypertensive agent should not disrupt the antitumor effect of Bev (12, 13). Instead, it is better to strengthen the antitumor effects of chemotherapies with antiangiogenic drugs (14).

Although Bev was found to affect tumor vascular normalization, it exacerbated more extracellular matrix (ECM) components such as collagen and hyaluronan (HA) deposition (15–19). ECM constitute an interstitial barrier, which can further increase solid stress and compress tumor vessels, ultimately leading to the insufficient perfusion of chemotherapeutic agents in tumor tissues (15, 20). Studies in animal models have shown that the deposition of collagen and HA can damage blood vessel function and reduce drug delivery to tumors (21, 22). Angiotensin system inhibitors (RASIs), which mainly include ACEIs and ARBs, have been confirmed to have therapeutic effects in fibrotic diseases of the lung, myocardium, and kidney (23). In the tumor-research fields, some studies have demonstrated that RASIs could improve survival benefits and reduce recurrence in colorectal cancer patients (24–26). RASIs have also been reported in tumor angiogenesis inhibition and matrix metalloproteinase-2 (MMP2) reduction (27, 28). On the contrary, other studies suggested that patients using ACEIs were associated with an increased risk of lung cancer and ARBs were associated with a modestly increased risk of new cancer diagnosis, which warrant further investigation (29–31).

We hypothesized when treating Bev-induced HT, RASIs could decrease ECM component remodeling induced by Bev, then improve drug delivery in solid tumors. A retrospective analysis of clinical data from mCRC patients was conducted to investigate which anti-hypertensive agent is more appropriate for Bev-induced HT in mCRC patients. Then, an experiment in HCT116 colon cancer cell xenografts in mice was explored to confirm the combined treatment with Bev and lisinopril (Lis), a RASI, could inhibit angiogenesis and ECM remodeling.

## Methods

### Retrospective Study Design

A retrospective study of patients diagnosed with mCRC who were treated with Bev and chemotherapeutics from January 2013 to January 2020 at the General Hospital of Northern Theater Command, China, was performed. The inclusion criteria were the presence of mCRC, ≥ 1 site of metastasis with no surgery, treatment with a combination of chemotherapy and Bev for ≥ 2 cycles, detailed medical records showing blood pressure, and antihypertensive treatment. The exclusion criteria were treatment with chemotherapy regimens that did not meet the inclusion criteria; an insufficient number of chemotherapy and targeted therapy cycles; incomplete medical records with no response evaluation or blood pressure measurement; treatment termination for psychological reasons; drug intolerance or other factors; and surgical resection during chemotherapy and antiangiogenic targeted therapy, local radiofrequency ablation or hepatic artery embolization. Eligible mCRC patients received 5 mg/kg Bev every 2 weeks or 7.5 mg/kg every 3 weeks in combination with 5-Fu or capecitabine ± oxaliplatin or irinotecan-based chemotherapy. The details of the retrospective study assessments, including patient enrollment, evaluation of treatment response, and hypertension, are shown in the **Supplementary Material**. The study was conducted according to the Declaration of Helsinki and approved by the Ethics Committee of the General Hospital of Northern Theater Command, China with informed consent waiver (no: Y2020046).

### Reagents and Antibodies

Bev injection (100 mg/4 mL, Avastin, Roche Pharma, Switzerland);Lis hydrochloride tablets (10 mg, Zhongfu, China); 5-Fu injection (0.25 g/mL, Xudong, China); 0.9% sodium chloride injection (100 mL, Baxter, China);Dulbecco’s modified Eagle’s medium (DMEM) and fetal bovine serum (FBS) (HyClone, UT, USA); a BCA protein quantification kit (Beyotime Biotechnology, Shanghai, China); an ECL detection kit, anti-TGF-β1 and anti-Collagen I (Wanleibio, Shenyang, China); anti-Smad2/3, anti-phospho-Smad2/3 and anti-Smad4 (Affinity Biosciences, OH, USA); anti-CD31 and anti-α-SMA (Servicebio, Wuhan, China); anti-GAPDH and secondary antibodies for Western blot analysis (Proteintech, Chicago, USA) were used.

### Cell Lines and Cell Culture

Colon cancer cell lines HCT116 were obtained from American Type Culture Collection (ATCC, Manassas, Virginia). The cells were cultured in DMEM supplemented with 1% penicillin-streptomycin and 10% FBS in a humidified environment at 37°C in a 5% CO_2_ atmosphere.

### Human Colorectal Cancer Xenograft Model

Female BALB/c-nu mice (4 – 6 weeks, weight 18 ± 2 g) were purchased from Beijing HFK Bioscience Co., Ltd., (China) and maintained in a special pathogen-free environment. The food and water were available ad libitum. The mice were maintained under a 12-h light/dark cycle at relative humidity 40–60% and room temperature 24 – 26 °C. All experimental procedures were carried out in accordance with the guidelines of the Animal Experimental Ethics Committee of the General Hospital of Northern Theater Command. After 5 days of acclimatization to the environment, each mouse was implanted with 0.2 mL of 5×10^6^ HCT116 cells/mL by subcutaneous injection into the unilateral axilla to establish one tumor. Tumor length and width were measured by calipers every two days, and the volumes were calculated with the following formula: V = (L × W^2^)/2, where V = the tumor volume (mm^3^), L = the length of the tumor along its major axis (mm), and W = the width of the tumor along its minor axis (mm). Drug administration began when the tumor volume was 50 –70 mm^3^.

### *In Vivo* Study Design

The mice were randomly divided into six groups (n = 12 in every group): the Con group (0.2mL PBS, po, per day), Lis group (2.5 mg/kg Lis, po, per day), low-dose Bev group (5 mg/kg Bev, ip, per 3 days), high-dose Bev group (10 mg/kg Bev, ip, per 3 days), Lis + low-dose Bev group (2.5 mg/kg Lis, po, per day combined with 5 mg/kg Bev, ip, per 3 days) and Lis + high-dose Bev group (2.5 mg/kg Lis, po, per day combined with 10 mg/kg Bev, ip, per 3 days). Dosage of Lis and Bev was calculated based on clinically dose conversion and previous studies (32, 33). Tumor volume and mouse weight were measured every 2 days until the end of the experiment on day 12. Tumors were randomly extracted on days 3 and 6 for the quantitative detection of VEGFA (n = 3 per time point). On day 12, the mice were intraperitoneally injected with 20 mg/kg 5-Fu and sacrificed 10 minutes later, after which the tumors were photographed and weighed (n = 6). Tumor samples were frozen at -80°C or fixed in paraformaldehyde for further analysis of 5-Fu concentration determination, immunofluorescence, ELISA, Sirius Red staining and western blot.

### Determination of the 5-Fu Concentration in Tumor Tissues

The 5-Fu concentration in tumor tissues was measured with a LC-MS/MS (LC21A, Shimadzu, Japan; Triple Quad™ 5500, SCIEX, USA) system. The 5-Fu reference standard and internal standard, 5-bromoutacil (5-Bu), were purchased from Victory Biological Technology Co., Ltd. (Sichuan, China). LC was carried with a Thermo Scientific™ Hypersil GOLD™ column (5 μm, 4.6 mm × 150 mm) and the following conditions: mobile phase: methanol-formic acid-deionized water (50: 0.1: 49.9), isocratic elution mode,10-minute elution time, 0.3 mL/min flow rate, 35°C column temperature, and a 5-μL injection volume. The mass spectrometer was equipped with an electro spray ionization (ESI) probe. The multiple reaction monitoring (MRM) m/z transitions monitored were 128.9 – 42.1 (CE – 33 V) for 5-Fu and 188.8 – 42.1 (CE - 37 V) for 5-Bu.

### Immunofluorescence Staining and Analyses

The paraformaldehyde-fixed tumor tissues were embedded in paraffin and cut into sections (5 μm) with a slicer (KD-2508, Zhejiang Jinhua Kedi Instrumental Equipment CO., LTD, China). The paraffin sections underwent dewaxing, hydration, antigen retrieval, and serum blocking and were then incubated with anti-CD31 antibody (1:100) and anti-α-SMA antibody (1:500) at 4°C overnight. Cy3 goat anti-rabbit IgG (1:300) and 488 goat anti-mouse IgG (1:400) were then added. After the nuclei were counterstained with DAPI and auto fluorescence had been quenched, the stained tissues were observed and imaged by inverted fluorescence microscopy (Olympus BX53). Morphological observation and quantitative analysis were conducted with Image J software (version 1.49, National Institutes of Health, Bethesda, MD, USA). Nine optical fields of CD31^+^hot spots per tumor section were selected in high-power vision (×200). The MVD was calculated by counting the number of individual CD31-positive luminal structures in each field. The VMI was calculated by determining the ratio of areas doubly positive for CD31 and α-SMA in each field according to the previous studies (34–36).

### VEGFA and HA ELISAs of Tumor Tissues

Tumor tissues were weighed, cut into small pieces, homogenized in cold PBS (pH 7.4) and centrifuged at 3,000 ×*g* for 20 minutes at 4°C to obtain supernatant samples. Protein expression in the tumor tissues was measured with ELISA kits for VEGFA (Cloud-Clone Corp, China) and HA (Mskbio, China) according to the manufacturers’ instructions.

### Evaluation of Collagen in Tumor Tissues

Collagen deposition in tumor tissues was investigated by Sirius Red staining. Paraffin sections were dewaxed and hydrated *via* graded ethanol (70%, 85%, 95%, and 100%, v/v) before being stained with Weigert’s iron hematoxylin solution for 10–20 minutes. Following differentiation with acidic differentiation solution and washing with distilled water, the sections were stained with Sirius red staining solution for 1 hour, gently rinsed, dehydrated with ethanol, cleared with xylene, and gradually mounted with neutral gum. Finally, the slices were imaged with an ordinary optical microscope and a polarized optical microscope.

### Protein Extraction and Western Blot Analysis

Tumor tissues were lysed with RIPA lysis buffer (Beyotime Biotechnology, Shanghai, China). Protein concentrations were detected by BCA protein assay kits (Beyotime Biotechnology, Shanghai, China). Proteins (50 µg/lane) were electrophoretically separated on 12% SDS-PAGE gels and transferred to PVDF membranes. The membranes were immuneblotted with specific primary antibodies and then incubated with HRP-conjugated secondary antibody. HRP was detected with an ECL system (Beyotime Biotechnology, Shanghai, China). The resultant bands were imaged by a ChemiDoc Touch Imaging System (Bio-Rad Laboratories, Inc., USA) and quantified by Image J software.

### Statistical Analyses

Datasets were analyzed by Student’s t-test or ANOVA, and the results are shown as the mean ± SEM. Statistical significance was defined at *P* < 0.05. For the retrospective analysis, the Pearson’s chi-square test or Fisher’s exact test was used to assess differences between the groups. progression-free survival (PFS) and overall survival (OS) curves were estimated using the Kaplan-Meier method, while the associations between survival time and predictor variables were statistically investigated using the Cox proportional hazards regression model. All calculations were performed using IBM SPSS Statistics 25.0 (SPSS, Inc., Chicago, IL, USA) and GraphPad Prism 7.0 (GraphPad Software, Inc., San Diego, CA, USA).

## Results

### Patient Characteristics in the Retrospective Study

A retrospective analysis of 94 patients with mCRC was conducted to investigate the optimal choice of anti-hypertensive agent for the treatment of Bev-induced HT. Eligible patients received Bev in combination with 5-Fu or capecitabine ± oxaliplatin or irinotecan-based chemotherapy were included: 63 males (67.02%) and 31 females (32.98%). The median age was 58 years. For forty-four patients (46.81%), the primary lesion was in the rectum, and for fifty patients (53.19%), it was in the colon. Fifty-six patients (59.57%) underwent surgical resection before chemotherapy and Bev therapy. Twenty-five patients (26.60%) had a history of HT controlled with antihypertensive drugs.

The patients were divided into a Bev-induced HT (Bev-HT) group (n = 41) and a non-HT group (n = 53) based on blood pressure measurements and assessment criteria (**Supplementary Table 1**). There were no differences between the two groups in age, sex, Karnofsky Performance Status (KPS), pathological tissue genotype, primary lesion location, or comorbidities including hypertension and coronary heart disease. The Bev-HT group included more patients with diabetes (22.00% *vs.* 13.2%, *P* = 0.03).

**Figure 1:**
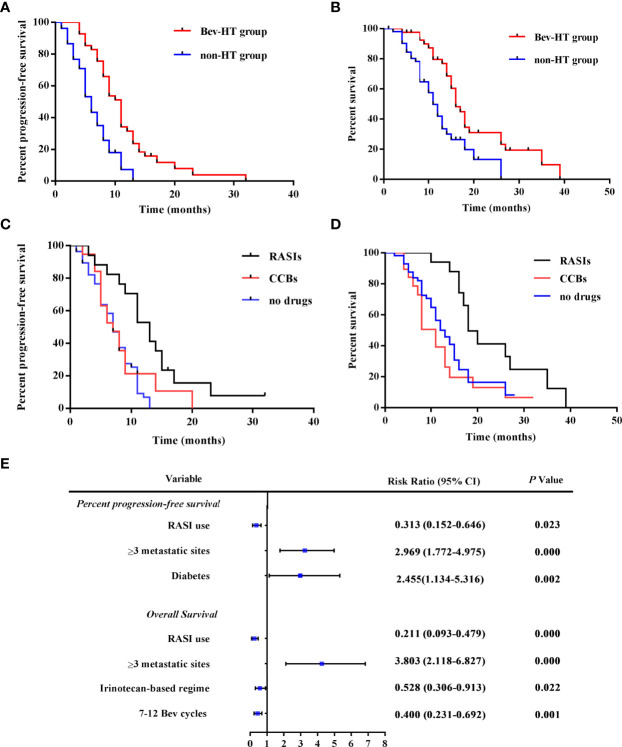
RASI adjunctive treatment improves survival in patients with mCRC. **(A)** PFS curves according to hypertensive status. **(B)** OS curves according to hypertensive status. **(C)** PFS curves according to antihypertensive treatment. **(D)** OS curves according to antihypertensive treatment. **(E)** The results of multivariate analysis of patient characteristics. HT, hypertension; RASIs, renin-angiotensin system inhibitors; CCBs, calcium channel blockers; SBP, systolic blood pressure; DBP, diastolic blood pressure; Bev, bevacizumab; 95% CI, 95% confidence interval.

In the Bev-HT group, both systolic blood pressure (SBP) and diastolic blood pressure (DBP) increased significantly after Bev treatment (SBP, 125.2 ± 11.54 mmHg *vs*. 154.6 ± 12.89 mmHg; DBP, 79.15 ± 7.02 mmHg *vs*. 94.88 ± 8.34 mmHg, *P<*0.001). According to the Common Terminology Criteria for Adverse Events version 5.0 (CTCAE v5.0) (8), 27 patients in the Bev-HT group were classified as developing grade 2 HT, and 14 patients developed grade 3 HT, but no patients developed grade ≥ 4 HT. PFS and OS were found to significantly differ between the Bev-HT group and non-HT group [median PFS 10.5 *vs.* 5.5 mo, hazard ratio (HR) 0.3049, 95% confidence interval (*CI*) 0.1809 – 0.514, *P* < 0.0001; median OS 16.0 *vs.* 11.0 mo, HR 0.3798, 95% *CI* 0.2161 – 0.6676, *P* = 0.0008] (**Figures 1A, B**).

### Adjunctive Treatment With RASIs Improves Survival in Patients With mCRC

Then, subgroups according to different antihypertensive treatment were divided for the further analysis. Compared with patients who received CCBs (n = 19), patients who received RASIs (n = 17) had a longer PFS and OS (median PFS 13.00 *vs.* 7.0 mo, HR 0.3961, 95% *CI* 0.1753 – 0.8951, *P* = 0.0260; median OS 18.00 *vs.* 11.00 mo, HR 0.2514 95% *CI* 0.1066 – 0.5929, *P* = 0.0016). Moreover, we observed a significant larger improvement in PFS and OS in the patients who received RASIs than the patients who did not receive any antihypertensive drugs (n = 58) (median PFS, 13.00 *vs.* 7.00 mo, HR 0.2870, 95% *CI* 0.1579 – 0.5214, *P* < 0.0001; median OS 18.00 *vs.* 13.00 mo; HR 0.3708, 95% *CI* 0.1992 – 0.6902, *P* = 0.0018) (**Figures 1C, D**). The result of multivariate regression analysis showed that RASI use is a significant factor for the improvement of PFS and OS. Moreover, irinotecan-based chemotherapy and 7–12 cycles of Bev treatment were found to be independently predictive of improved OS, while diabetes and ≥ 3metastatic sites were risk factors for poor prognosis in the patients (**Figure 1E**). The results of this retrospective study indicated that when treating Bev-HT, RASIs might improve survival in patients with mCRC.

### Bev and Lis Synergistically Inhibited the Growth of HCT116 Xenograft Tumors in Mice

**Figure 2:**
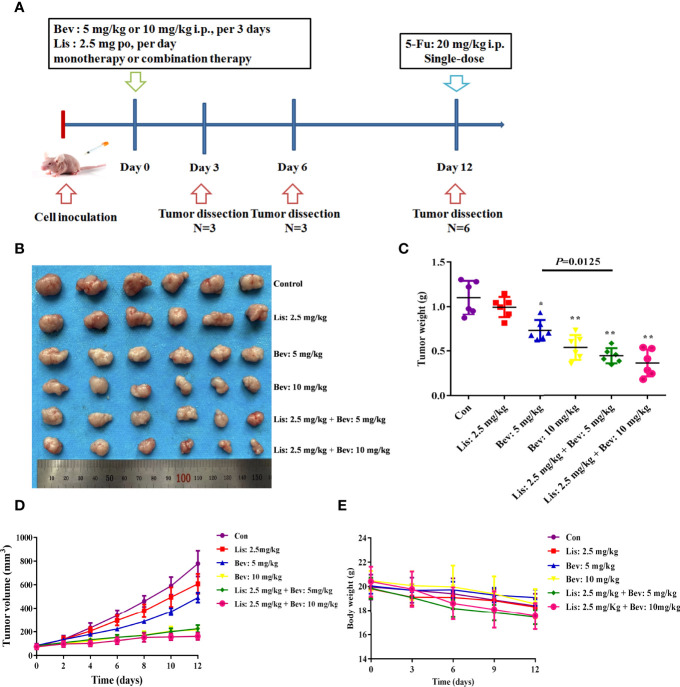
Bev and Lis synergistically inhibit tumor growth in HCT116 xenografts. **(A)** Schematic of the study design. BALB/c nude mice were subcutaneously injected in the bilateral flank with HCT116 cells. The mice were treated with Bev at 5 mg/kg or 10 mg/kg by intraperitoneal injection every 3 days and/or Lis at 2.5 mg/kg orally once daily. **(B)** Images of tumors excised from the mice (n = 6). **(C)** Means of the tumor weights. **(D)** Tumor growth curves were generated by measuring tumor volumes every 2 days. **(E)** Means of the mouse weights. Data represent the mean ± SEM. *P< 0.05, **P< 0.01 *versus* the control group. Con, control; Bev, bevacizumab; Lis, lisinopril; 5-Fu, 5-fluorouracil.

A human HCT116 colon cancer cell xenograft mouse model was established, and the inhibitory effect on tumor volume of the angiotensin-converting enzyme inhibitor (ACEI) Lis and Bev on subcutaneous tumors was investigated. The mice were randomly divided into six groups and treated according to dose-conversion between mice and humans and previous studies (32, 33):

A schematic of the study design is shown in **Figure 2A**. After treatment with Bev monotherapy (5 mg/kg and 10 mg/kg), tumors resolved rapidly and lastingly, which was significant and dose-dependent (**Figures 2C, D**). Although Lis monotherapy tended to slow tumor growth compared with the Con condition, this difference was not statistically significant. Notably, combination treatment with Bev and Lis significantly enhanced the inhibition of tumor growth induced by Bev monotherapy (**Figures 2B–D**). According to the formula of drug combination q = E (A + B)/(EA + EB – EA × EB) (37, 38), Bev and Lis were determined to synergistically suppress tumor growth (q = 1.41). The weight of the mice in each group decreased throughout the 12 days of the experiment, with no significant difference among the groups (**Figure 2E**). Thus, the results of these *in vivo* experiments indicated that the synergistic therapeutic effects of Bev and Lis are better than the effect of single-agent antitumor therapy.

### Bev and Lis Synergistically Increased 5-Fluorouracil (5-Fu) Delivery to Tumor Tissues

**Figure 3:**
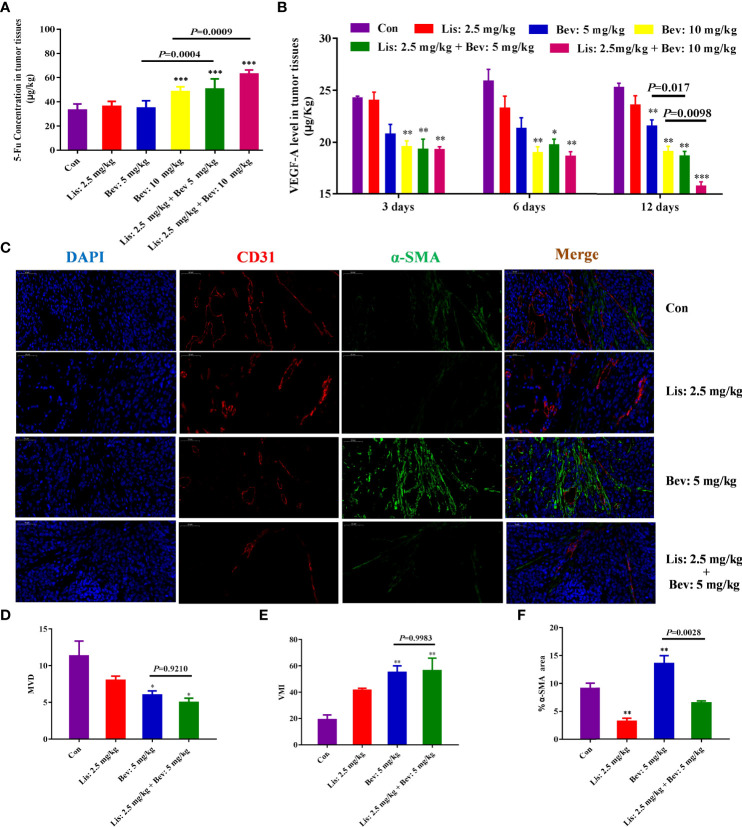
Synergistic effects of Bev and Lis on 5-Fu tissue penetration and angiogenesis in HCT116 xenografts. **(A)** Synergistic effect of Bev and Lis on 5-Fu concentration in tumor tissues (n = 6). **(B)** VEGFA levels in tumor tissues at 3, 6, and 12 days after Lis and Bev administration. **(C)** Immunofluorescence staining images of CD31 and α-SMA (Scale bar = 50μm). **(D)** The effects of Bev and Lis on MVD. **(E)** The effects of Bev and Lis on VMI. **(F)** The proportions of α-SMA-positive areas were quantified using ImageJ software. Data represent the mean ± SEM. *P< 0.05, **P< 0.01, ***P< 0.001 *versus* the control group. Con, control; Bev, bevacizumab; Lis, lisinopril; 5-Fu, 5-fluorouracil; VEGFA, vascular endothelial growth factor A; MVD, microvessel density; VMI, vascular maturity index.

To study the penetration of chemotherapy drugs, we then determined the concentration of 5-Fu in xenograft tumors by liquid chromatography tandem mass spectrometry (LC-MS/MS). Bev monotherapy (10 mg/kg) and the addition of Lis (2.5 mg/kg) significantly increased the concentration of 5-Fu in tumor tissues compared with that of the Con group (**Figure 3A**). In particular, tumor tissues in the combination therapy groups showed higher 5-Fu concentrations than those in the Bev monotherapy groups. The results indicated that Bev and Lis synergistically increased 5-Fu delivery to tumor tissues

### Antiangiogenic Effects of Bev and Lis

VEGFA, the primary member of the VEGF family, directly functions in the proliferation and permeability of endothelial cells and induces physiological angiogenesis in mCRC (39). Therefore, to study the angiogenesis effects of Bev and Lis, we investigated the levels of VEGFA in the xenograft tumor tissues of mice in the different treatment groups at different time points. After 3 and 6 days of treatment, compared with the Con group and Lis monotherapy group, the 10 mg/kg Bev monotherapy group and the two Lis and Bev combination groups inhibited VEGFA levels significantly (**Figure 3B**). Moreover, as the treatment was prolonged to 12 days, the two combination groups showed dose-dependent significant differences in decreasing VEGFA compared with the Bev monotherapy groups. The results indicated that combined treatment with Bev and Lis could synergistically inhibit VEGFA, which inhibit angiogenesis (**Figure 3B**).

Anti-angiogenic therapies, which ‘normalize’ the abnormal blood vessels in tumors, have been proven to improve the delivery and effectiveness of chemotherapeutics (40). The synergistic effects of Bev and Lis on tumor blood vessel normalization were investigated by CD31 and α-SMA immunofluorescence double staining (**Figure 3C**). Compared with 5 mg/kg Bev, 2.5 mg/kg Lis did not contribute to the effects of Bev on MVD or VMI in tumor tissues, which confirmed that Lis did not disrupt the ability of Bev to normalize the vasculature (**Figures 3D, E**). α-SMA is the gold standard marker of fibroblast activation (41). Compared with the Con group and the 5 mg/kg Bev group, the Lis group showed a significant decrease in fibroblasts, as demonstrated by α-SMA staining (*P*=0.0028). These findings suggest that Lis prevents the fibrotic infiltration of tumor tissues (**Figure 3F**).

### Lis Reduces Extracellular Matrix (ECM) Component Remodeling by Bev

**Figure 4:**
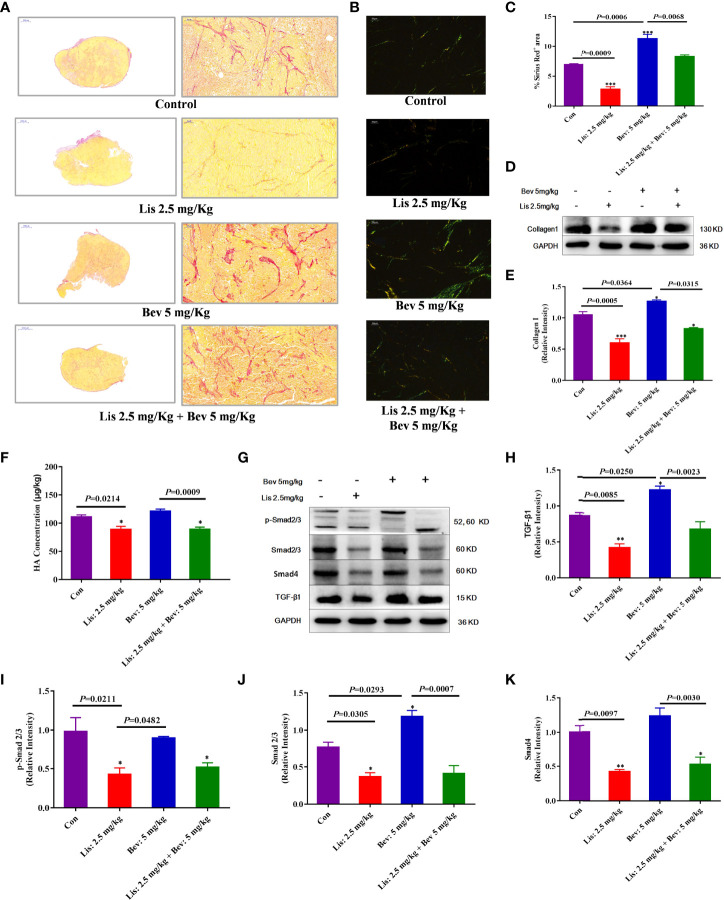
Lis reduced ECM remodeling by Bevin HCT116 xenograft models. **(A)** Collagen staining by Sirius Red and imaging by light microscopy (Scale bar = 1000μm and 50μm). **(B)** Collagen staining by Sirius Red and imaging by polarized light (Scale bar = 50μm). **(C)** The proportions of Sirius Red-positive areas were quantified using Image J software. For each staining experiment, with three sections per tumor (n = 3). **(D)** Western blot analysis of collagen I in tumor tissues. **(E)** Quantitative collagen I protein expression (n = 3). **(F)** ELISA of the HA expression level (n = 6). **(G)** Western blot analysis of the protein expression of ECM markers in tumor tissues. **(H–K)** Quantitative protein expression levels. Data are the mean ± SEM. *P< 0.05, **P< 0.01, ***P< 0.001 *versus* the control group. ECM, extracellular matrix; Con, control; Bev, bevacizumab; Lis, lisinopril; HA, hyaluronic acid; TGF-β1,transforming growth factor β1.

Collagens, fibronectins and proteoglycans, including HA, are the primary ECM components in colon cancer tissue, and the ECM level increases during the malignant pathological process (42–44). Collagen I expression after Bev and Lis intervention was investigated by Sirius Red staining and Western blot assays. The results indicated that compared with the control treatment, 5 mg/kg Bev treatment significantly increased collagen fiber deposition and collagen I protein expression, while 2.5 mg/kg Lis treatment decreased these parameters (**Figures 4A–E**). The results of enzyme-linked immunosorbent assay (ELISA) showed that compared with the Con condition, Lis monotherapy significantly decreased the expression level of HA (*P* = 0.0214). Furthermore, compared with Bev treatment, Lis treatment significantly reduced the HA level in tumor tissues (*P*= 0.0009) (**Figure 4F**). The combination therapy of Bev and Lis significantly decreased the collagen and HA level compared with Bev monotherapy. These results implied that Lis might reverse Bev-induced ECM component remodeling.

### Lis Inhibits ECM Remodeling Induced by Bev by Downregulating the TGF-β1/SMAD Signaling Pathway

ECM remodeling results in interstitial barriers and induces insufficient perfusion, which can be exacerbated by antiangiogenic treatment (45, 46). To clarify the underlying mechanisms by which Lis reversed Bev-induced ECM remodeling, we further evaluated the protein expression of TGF-β1, an ECM-related signaling pathway marker (47). Compared with that in the Con group, the expression level of TGF-β1 was significantly decreased in the Lis group; moreover, Lis inhibited the Bev-induced increase in TGF-β1 expression (**Figures 4G, H**). TGF-β1 promotes tissue fibrosis, which triggers downstream SMAD signaling pathways, and participates in ECM synthesis and deposition during biological processes (48). Compared with the Con group and 5 mg/kg Bev group, the 2.5 mg/kg Lis group significantly decreased total-Smad2/3, phosphorylated-Smad2/3, and Smad4 protein expression (**Figures 4I–K**). These findings indicated that 2.5 mg/kg Lis might inhibit ECM remodeling induced by Bev through downregulating the expression of components in the TGF-β1/SMAD2/3 signaling pathway.

## Discussion

To investigate an appropriate antihypertensive treatment for Bev-induced HT and explore the synergistic anti-tumor effect of antihypertensive agents and Bev, we firstly conducted a retrospective analysis of the survival benefits in different antihypertensive drugs on patients with mCRC who were treated with Bev and standard chemotherapy. Compared with those who received CCBs, patients who received ACEIs or ARBs had a longer PFS and OS. Then, we performed an *in vivo* study of HCT116 colon cancer xenografts in mice and confirmed that 5 mg/kg or 10 mg/kg Bev and 2.5mg/kg Lis synergistically inhibited tumor growth and increased the 5-Fu concentration in tumor tissues. The findings indicated that RASIs might be the optimal choice for Bev-induced HT in the treatment of mCRC.

Our study demonstrated that Lis monotherapy tended to inhibit tumor growth and VEGFA expression, but compared with the control group, the difference was not statistically significant. Moreover, the addition of Lis did not contribute to effects of Bev on MVD or the VMI, which indicated that Lis did not directly promote the penetration of 5-Fu, a chemotherapeutic drug, by vascular normalization. Instead, Lis might have cooperated with Bev through another mechanism to increase the concentration of 5-Fu in tumors. Previous research has suggested the vascular normalization and solid stress alleviation treatments are promising strategies to improve tumor perfusion and delivery of drugs (49). Although Bev improved tumor vascular normalization, it showed a significant increase in fibroblasts of tumor tissues, as demonstrated by α-SMA staining, and exacerbated collagen and HA deposition, which could damage blood vessel function and reduce drug delivery to tumors. Our results suggest Lis could reverse ECM component enhanced by Bev.

Subsequent experiments suggested that Lis inhibits TGF-β1, a multifunctional cytokine associated with ECM component remodeling, tumor angiogenesis, invasion, and metastasis (50). Furthermore, TGF-β1 levels were previously shown to be significantly increased and associated with poor clinical prognosis in colorectal cancer (51, 52). TGF-β subfamily signaling begins with TGF-β ligand activation and binding to Ser/Thr kinase receptors, which in turn causes phosphorylation of the downstream target Smad2/3. Phosphorylated Smad2/3 forms a heterodimeric complex with Smad4 that then translocates to the nucleus (53). Our results showed that the expression of TGF-β1 and downstream SMAD signaling components was enhanced by Bev, ultimately remodeling primary ECM components, whereas Lis inhibited these effects.

This study has some limitations. Firstly, due to the limited number of patients in the retrospective study, we did not establish subgroups to further evaluate whether there were differences in survival benefits between patients treated with ACEIs and ARBs. Studies indicated that angiotensin-II-receptor-1 (AT1) and angiotensin-II-receptor-2 (AT2) signaling have opposite effects on tumor fibrosis; thus, ACEIs reduced collagen I and hyaluronan to a lesser extent than ARBs (54, 55). However, Rahbari *et al.* demonstrated that AT1 deletion did not affect HA expression, and the abnormal deposition of ECM observed after anti-VEGF therapy was independent of the AT1 pathway (15). HT of all grades has been observed in up to 36% of patients treated with Bev, and the reported incidence of high-grade HT ranges from 1.8% to 22%, with up to 1% of events being grade 4 in clinical trials (4). Considering the intensity of its antihypertensive effect and long-term cardioprotection, ACEI treatment is more likely to reduce stroke, nonfatal myocardial infarction, and cardiovascular and total mortality in high-risk patients than ARB treatment (56). Secondly, we did not further investigate the effect of Bev on blood pressure fluctuations and the underlying mechanisms of Bev-induced hypertension in mice. The pathological and physiological mechanisms of Bev-HT are still complicated, including inhibition of nitric oxide synthase, injury of the vascular endothelial system, decrease of capillary density, oxidative stress, et al. (4, 7), which are worthy of in-depth study in the future. Thirdly, the traditional subcutaneous tumor model was used in this study, which might be less suitable for studying most aspects of the tumor microenvironment (57). Orthotopic mouse models of colorectal cancer, which could feature cancer cells growing tumors in their natural location, simulate tumor microenvironment better and replicate human disease with high fidelity (58–60). Last but not least, the effects of different RASIs in combined with different doses of Bev on cancer-associated fibroblasts and ECM components through *in vitro* studies should also to be further explored.

In summary, mCRC patients who received RASIs were found to have a better survival prognosis than the patients who received CCBs. The combination of Bev and Lis might synergistic inhibit angiogenesis and ECM component deposition, which promoted 5-Fu perfusion into the tumor. Thus, RASIs might be an optimal choice for Bev-induced HT.

## Data Availability Statement

The original contributions presented in the study are included in the article/**Supplementary Material**. Further inquiries can be directed to the corresponding authors.

## Ethics Statement

The studies involving human participants were reviewed and approved by the Ethics Committee of the General Hospital of Northern Theater Command, China (no: Y2020046). Written informed consent for participation was not required for this study in accordance with the national legislation and the institutional requirements. The animal study was reviewed and approved by the guidelines of the Animal Experimental Ethics Committee of the General Hospital of Northern Theater Command.

## Author Contributions

TR, YSZ, and QZ conceived of and designed the research. TR, HJ, QW, QM, DY, DX, and ZX performed the research. TR, YZ, and HJ analyzed the data and wrote the manuscript. YSZ, HJ, XG, and QZ reviewed the manuscript. All authors have made an intellectual contribution to the manuscript and approved its submission.

## Funding

This study was supported by a Liaoning Science and Technology Research Project (no. 2020-BS-125).

## Conflict of Interest

The authors declare that the research was conducted in the absence of any commercial or financial relationships that could be construed as a potential conflict of interest.

## Publisher’s Note

All claims expressed in this article are solely those of the authors and do not necessarily represent those of their affiliated organizations, or those of the publisher, the editors and the reviewers. Any product that may be evaluated in this article, or claim that may be made by its manufacturer, is not guaranteed or endorsed by the publisher.
