# Supplementary material for: Inhibition of Angiogenesis and Extracellular Matrix Remodeling: Synergistic Effect of Renin-Angiotensin System Inhibitors and Bevacizumab
Source: Front Oncol. 2022 Jul 1;12:829059. doi: 10.3389/fonc.2022.829059 (PMC9283643; doi:10.3389/fonc.2022.829059)
Supplement: Supplementary file 1 [file Table_1.docx]

**Supplimentary material**

**Inhibition of Angiogenesis and Extracellular Matrix Remodeling: Synergistic Effect of Renin-Angiotensin System Inhibitors and Bevacizumab**

**Running header:**

**Inhibition of angiogenesis and ECM by Bev and RASIs**

Tianshu Ren^a,b^, Hui Jia ^b^, Qiong Wu^b^, Yan Zhang^b^, Qun Ma^b^, Dong Yao^b^, Xudong Gao^b^, Danni Xie^b^, Zihua Xu^b^, Qingchun Zhao^a,b *^, Yingshi Zhang^a**^

**Retrospective Study Assessments**

Patient and tumor characteristics, including age, sex, concomitant chronic disease, KPS, location of primary lesion, metastasis location and number, TNM stage, diagnosis time, recurrence time and death time, chemotherapy and targeted treatment, were recorded in detail and were obtained from medical record for the purposes of this study.

The criteria for the Bev-induced hypertension (HT) group included initiation of an antihypertensive agent after the first treatment with Bev, an increase in a preexisting antihypertensive medication or the addition of a new antihypertensive agent while receiving therapy, or at least 2 consecutive documented blood pressure measurements of > 140 / 90 mmHg after the initiation of therapy in patients with no prior record of hypertension. For the patients with a history of hypertension with an increase in the antihypertensive agent dose or a new antihypertensive medication prescription on the basis of the original antihypertensive regimen were included in the Bev-HT group. For patients with a documented history of hypertension and blood pressure fluctuations were stable during Bev treatment who did not require an increase in antihypertensive agents were included in the “non-HT” group[[1](#_ENREF_1)]. Peripheral blood pressure was measured using Yuwell YE680B upper-arm blood pressure monitor (YuYue, Jiangsu Province, China) and recorded by the nurses during hospitalization, including before and after bevacizumab (Bev) infusion.

The highest arterial blood pressure value recorded after the first administration of Bev during hospitalization was taken into account to define the grade of Bev-induced HT according to the Common Terminology Criteria for Adverse Events version 5.0 (CTCAE v5.0) [[2](#_ENREF_2)] but adapted for this study as follows[[3](#_ENREF_3), [4](#_ENREF_4)]: grade 1: asymptomatic, transient (<24h) increase of > 20 mmHg (diastolic blood pressure, DBP) or to 120-139 mmHg (systolic blood pressure, SBP) if previously within normal limits, no treatment required; grade 2: recurrent or persistent increase of > 20 mmHg (DBP) or to 140～159 mmHg (SBP) if previously within normal limits, monotherapy with antihypertensive agents may be indicated; grade 3: increase of > 20 mmHg (DBP) or >160 mmHg (SBP), hypertension requiring more than one drug or more intensive therapy than previously; grade 4: life-threatening consequences (e.g. malignant hypertension, transient or permanent neurologic deficit, hypertensive crisis); urgent intervention indicated.

Treatment response was evaluated according to Response Evaluation Criteria In Solid Tumors (RECIST) by whole body spiral computerised tomography performed every 8 weeks. Clinical outcomes were defined as progression-free survival (PFS) and overall survival (OS). PFS was the time from the date of initial Bev therapy to the first documented occurrence of disease progression or death due to any cause. OS was defined as the time from the start of Bev therapy to death due to any cause. Patients who were lost to follow-up or still alive were censored at the date of the last visit.

**Supplementary Table 1**

**Characteristics and univariate analysis of mCRC patients treated with Bev**

| **Characteristic** | **HT group**  **(n=41)** | **non-HT group**  **(n=53)** | ***P* value** | **Univariate analysis of**  **PFS** | **Univariate analysis of**  **OS** |
| --- | --- | --- | --- | --- | --- |
| **Median age, years (range)** | 60 (50–69) | 57 (46–67) | 0.120 | 0.148 | 0.045 |
| **Sex, n (%)** |  |  |  |  |  |
| Male | 25 (61.0) | 38 (71.70) | 0.273 | 0.541 | 0.984 |
| Female | 16 (39.0) | 15 (28.30) |  |  |  |
| **Karnofsky performance status** | | | | |  |
| 0 | 15 (36.6) | 16 (30.2) | 0.513 | 0.353 | 0.524 |
| 1 | 18 (43.9) | 30 (56.6) | 0.222 | 0.377 | 0.376 |
| 2 | 8 (19.5) | 7 (13.2) | 0.408 | 0.976 | 0.681 |
| **Comorbidity(previous or current), n (%)** | | | |  |  |
| Hypertension | 12 (29.3) | 13 (24.5) | 0.606 | 0.073 | 0.112 |
| CHD | 5 (12.2) | 3 (5.7) | 0.290 | 0.364 | 0.569 |
| Diabetes | 9 (22.00) | 7 (13.2) | 0.263 | 0.030 | 0.314 |
| **Primary lesion site, n (%)** |  |  |  |  |  |
| Rectum | 20 (48.78) | 24 (45.28) | 0.736 | 0.922 | 0.759 |
| Colon | 21 (51.22) | 29 (54.72) |  |  |  |
| ***KRAS* status (wt: mutated), n (%)** | 7 (17.1): 10 (24.4) | 10 (18.9): 7 (13.2) | 0.376 | 0.692 | 0.810 |
| ***NRAS* status (wt: mutated), n (%)** | 10 (24.4): 2 (4.9) | 12 (22.6): 0 (0) | 0.253 | 0.778 | 0.919 |
| ***BRAF* status (wt: mutated), n (%)** | 12 (29.3): 1 (2.4) | 8 (15.1): 2 (3.8) | 0.244 | 0.243 | 0.363 |
| **Resection of primary tumor, n (%)** | 25 (61.0) | 31 (58.5) | 0.808 | 0.628 | 0.913 |
| **Number of metastatic sites, n (%)** |  |  |  |  |  |
| 1 | 21 (51.2) | 15 (28.3) | 0.023 | 0.151 | 0.079 |
| 2 | 10 (24.4) | 16 (30.2) | 0.533 | 0.807 | 0.378 |
| ≥3 | 10 (24.4) | 22 (41.5) | 0.082 | <0.001 | <0.001 |
| **Metastatic organs, n (%)** |  |  |  |  |  |
| Lung | 18 (43.9) | 28 (52.8) | 0.391 | 0.565 | 0.593 |
| Liver | 30 (73.2) | 38 (71.7) | 0.874 | 0.931 | 0.374 |
| Lymph | 13 (31.7) | 21 (39.6) | 0.428 | 0.921 | 0.915 |
| **Bev combined regimes, n (%)** |  |  |  |  |  |
| Oxaliplatin based | 29 (70.7) | 27 (50.9) | 0.053 | 0.014 | <0.001 |
| Irinotecan based | 8 (19.5) | 22 (41.5) | 0.023 | <0.001 | <0.001 |
| 5-Fu monotherapy | 4(9.8) | 4 (7.5) | 0.725 | 0.527 | 0.589 |
| **Median Bev cycles, n (%)** |  |  |  |  |  |
| 2–6 cycles | 20 (48.8) | 35 (66.0) | 0.092 | 0.745 | 0.072 |
| 7–12 cycles | 17 (41.5) | 18 (34.0) | 0.456 | 0.610 | 0.004 |
| <12 cycles | 4 (9.8) | 0 (0) | 0.033 | 0.151 | 0.151 |
| **Antihypertensive therapy** |  |  |  |  |  |
| RASIs | 14 (34.1) | 3 (5.7) | <0.001 | 0.008 | 0.004 |
| CCBs | 12 (29.3) | 7 (13.2) | 0.054 | 0.998 | 0.674 |
| No antihypertensive drugs | 15 (36.6) | 43 (81.1) | <0.001 | 0.016 | 0.017 |
| **Grade of Bev-induced HT** | | | | | |
| Grade 2 | 27 (65.9) |  |  | 0.009 | 0.016 |
| Grade 3 | 14 (34.1) |  |  | 0.388 | 0.315 |
| mCRC, metastatic colorectal cancer; HT, hypertension; CHD,coronary heart disease; RASIs, renin angiotensin system inhibitors; CCBs, calcium channel blockers. | | | | | |

**References**

1. Zhong J, Ali AN, Voloschin AD, Liu Y, Curran WJ, Crocker IR, et al. Bevacizumab-induced hypertension is a predictive marker for improved outcomes in patients with recurrent glioblastoma treated with bevacizumab. Cancer. 2015;121(9):1456-62.

2. Institute NC. Common Terminology Criteria for Adverse Events Version 5.0 published November 27, 2017. Retrieved from <http://ctepcancergov/reporting/ctchtml>. 2017.

3. Nakaya A, Kurata T, Yokoi T, Iwamoto S, Torii Y, Katashiba Y, et al. Retrospective analysis of bevacizumab-induced hypertension and clinical outcome in patients with colorectal cancer and lung cancer. Cancer medicine. 2016;5(7):1381-7.

4. Österlund P, Soveri LM, Isoniemi H, Poussa T, Alanko T, Bono P. Hypertension and overall survival in metastatic colorectal cancer patients treated with bevacizumab-containing chemotherapy. British journal of cancer. 2011 Feb 15;104(4):599-604.
